# Awareness and knowledge of impression techniques for cleft lip and palate among dentists: A cross-sectional survey

**DOI:** 10.6026/973206300212518

**Published:** 2025-08-31

**Authors:** Vidhyadhar D., Tejosmita Chowdary Pavuluri, Vivek Anne, Hanuraj Kumar Lankalapalli S.S., Vivek Buddha, Gona Maheshwar Reddy, Naseemoon Shaik

**Affiliations:** 1 Department of Dentistry, Kamareddy Government Medical College, Kamareddy, Telangana, India; 2 Department of Biomedical Science, Rutgers Biomedical and Health Sciences, Newark, New Jersey, USA; 3 Department of Epidemiology, Laramie Pediatrics & Internal Medicine pc, Wyoming, USA; 4 Department of Prosthodontics & Crown and Bridge, Lenora Institute of Dental Sciences, Rajanagaram, Rajamahendravaram, Andhra Pradesh, India; 5 Orofacial pain specialist, Family Dental of Virginia, USA; 6 Department of Prosthodontics & Crown and Bridge, Sri Balaji Dental College and Hospital, Moinabad, Telangana, India; 7 Department of Pedodontics and Preventive Dentistry, MNR Dental College and Hospital, Sangareddy, Telangana, India

**Keywords:** Cleft lip and palate, impression techniques, general dentists, digital, survey

## Abstract

Management of cleft lip and palate (CLP) requires precise impression techniques, yet the awareness and application of these methods among general dental practitioners in India remain underexplored. In this cross-sectional survey of 380 dentists using a validated 18-item questionnaire, 64% were familiar with conventional approaches, but only 20% knew of digital techniques. Urban practitioners showed significantly higher knowledge scores than rural counterparts (72 ± 9 vs. 61 ± 11; p<0.01), while just 30% felt confident using digital methods. Lack of training (70%) and limited access to equipment (58%) were the main barriers and only half recognized procedural risks such as compromised neonatal nasal breathing. These findings reveal substantial knowledge gaps, underscoring the need for targeted educational initiatives to enhance CLP management skills.

## Background:

Cleft lip and palate (CLP) is a prevalent congenital craniofacial anomaly, occurring in approximately 1 in every 700 live births worldwide. This condition poses significant challenges in feeding, speech, hearing, dentofacial development and psychosocial well-being. Early and precise intervention is crucial to improve functional and esthetic outcomes in affected individuals. A key component in the management of CLP involves the fabrication of specialized feeding appliances and pre-surgical orthopedic devices, which rely heavily on accurate impression techniques to capture the unique oral anatomy of neonates and infants. These impressions serve as the foundation for constructing nasoalveolar molding (NAM) appliances and other prosthetic devices that facilitate feeding and guide surgical planning [1, 2]. Conventional impression methods, such as alginate, putty-wash materials and NAM techniques, have been routinely employed in clinical practice due to their accessibility and familiarity among practitioners. However, these methods present certain limitations, including patient discomfort, risk of airway obstruction and potential inaccuracies when recording the delicate oral structures of neonates with clefts [3]. The introduction of digital intraoral scanning technologies has brought forth opportunities for more accurate, efficient and less invasive impression-making procedures. Digital workflows offer advantages such as enhanced precision, reproducibility, ease of data storage and improved patient comfort.

Despite these benefits, the application of digital impressions in CLP cases remains relatively limited due to the anatomical c omplexities presented by the cleft, the small size of neonatal oral cavities and the high cost and limited availability of advanced scanning equipment in many clinical settings [4]. General dentists often serve as the first point of contact for patients with CLP, either during routine examinations or through referrals. Their awareness and competence in selecting appropriate impression techniques are crucial for ensuring timely and accurate interventions. However, previous studies suggest that general dental practitioners may lack adequate training and exposure to specialized techniques required for managing CLP, particularly with regard to emerging digital technologies [5]. Limited access to resources, insufficient hands-on training opportunities and a lack of awareness about procedural risks, such as neonatal nasal breathing challenges during impression-making, further compound these barriers [4, 5]. Therefore, it is of interest to report the level of awareness, knowledge, and attitudes of general dentists in India regarding impression techniques for CLP management.

## Materials and Methods:

## Study design and population:

A cross-sectional survey was conducted between January and April 2025 among general dental practitioners across India. Participants were recruited through professional dental associations, online forums and social media platforms targeting dental professionals. A stratified sampling strategy was adopted to ensure balanced representation from urban and rural practice settings and varying levels of clinical experience (0-5 years, 6-10 years and more than 10 years).

## Data collection:

Data collection was conducted through an online survey administered via Google Forms. A snowball sampling technique was employed; initial participants were encouraged to share the survey with eligible colleagues to facilitate wider dissemination across diverse geographic regions and levels of clinical experience. The sample size was determined based on power analysis from previous studies, requiring a minimum of 380 participants to achieve sufficient statistical power. Accordingly, data collection was closed upon achieving the required sample size of 380 respondents. Participation was voluntary and anonymous. All data were stored securely and handled in accordance with ethical standards to ensure participant confidentiality.

## Questionnaire development:

A structured, self-administered 18-item questionnaire was developed to assess dentists' awareness, knowledge, attitudes and practices related to cleft lip and palate (CLP) impression techniques. The questionnaire captured demographic details (age, gender, practice setting and prior exposure to CLP-related procedures), awareness of conventional impression techniques (alginate, putty-wash, nasoalveolar molding [NAM]) and digital methods (intraoral scanner-based impressions), knowledge regarding procedural challenges such as neonatal nasal breathing difficulties and scanner limitations, attitudes toward the adoption of digital technologies and current utilization along with perceived barriers. Content validity was established through expert review and construct validity was confirmed via exploratory factor analysis. Internal consistency reliability was verified using Cronbach's alpha (α = 0.83), indicating good reliability.

## Statistical analysis:

Descriptive statistics were used to summarize demographic data, awareness levels, knowledge scores and attitudes. Associations between categorical variables (*e.g.*, knowledge and attitudes relative to demographic factors) were analyzed using Chi-square tests, with statistical significance set at p < 0.05. Differences in mean knowledge and attitude scores (scale: 0-100) between urban and rural dentists and across experience categories were assessed using independent samples t-tests.

## Results:

A total of 380 dental practitioners participated in the study. The majority of respondents were male (58%) and a substantial proportion practiced in urban areas (62%) (Table 1 - see PDF). Participants predominantly belonged to the 25-35 years age group (40%), followed by those aged 36-45 years (35%) and those above 45 years (25%). Regarding clinical experience, 36% had 0-5 years, 30% had 6-10 years and 34% had more than 10 years of practice. Notably, only 25% of respondents reported having received specific training related to cleft lip and palate (CLP) impression techniques, whereas 75% lacked such specialized training. The findings indicate that 64% (243/380) of dentists were aware of conventional CLP impression techniques. Specifically, awareness was highest for alginate impressions (60%), followed by putty wash (48%) and nasoalveolar molding (NAM) techniques (32%) (Table 2 - see PDF). However, awareness of digital intraoral scanning was limited to 20% of respondents. Regarding knowledge of procedural challenges, 45% correctly identified limitations with alginate, 36% with putty wash, 24% with NAM and 38% with digital scanning techniques. The mean knowledge score was 66 ± 11, with urban practitioners demonstrating significantly higher scores (72 ± 9) compared to rural counterparts (61 ± 11, p=0.001). Similarly, practitioners with over 10 years of experience showed greater knowledge (70 ± 10) than those with 0-5 years (62 ± 12, p=0.003). Awareness of neonatal nasal breathing risks was correctly identified by 50% of respondents, while 38% recognized the limitations of digital scanners in capturing deep alveolar clefts. Attitude scores, measured on a scale of 0-100, indicated an overall positive inclination towards CLP impression techniques, with a mean score of 68 ± 10 (Table 3 - see PDF). Urban practitioners reported significantly higher attitude scores (73 ± 8) than rural practitioners (62 ± 10, p<0.001). However, only 30% expressed confidence in adopting digital techniques. Among perceived benefits, accuracy (65%) and patient comfort (58%) were most frequently cited. The primary barriers to adoption were identified as lack of training (70%), limited access to equipment (58%) and high associated costs (42%). Practitioners with prior CLP-specific training exhibited more favorable attitudes (75 ± 7) compared to those without such training (65 ± 10, p<0.001).

## Discussion:

The present study highlights a moderate level of awareness (64%) among general dental practitioners regarding conventional cleft lip and palate (CLP) impression techniques, but reveals substantially lower awareness (20%) of digital intraoral scanning technologies. These findings are in alignment with previous studies reporting limited integration of digital dentistry (30-50%) within general dental practice settings [6, 7]. This trend reflects the broader challenges faced by general practitioners in keeping pace with rapidly evolving digital technologies that are more commonly adopted within specialized centers or academic environments. Notably, the higher knowledge scores observed among urban dentists (72 ± 9 vs. 61 ± 11, p=0.001) further corroborate earlier research underscoring the persistent urban-rural disparities in access to advanced technologies and continuing professional education [8, 9]. Urban practitioners often benefit from better exposure to newer technologies through workshops, conferences and collaborations with tertiary care centers, which may not be as readily available to their rural counterparts. However, the study also reveals that knowledge of procedural challenges, such as neonatal nasal breathing risks (50% awareness), remains limited among general dentists. This is in sharp contrast to the higher awareness levels (70-80%) reported in specialized CLP centers [10], underscoring a significant training gap in general practice settings. This gap is concerning given the critical importance of understanding these challenges to ensure safe and effective CLP care. The attitude scores (68±10) indicate a general sense of cautious optimism among respondents toward adopting digital techniques. Approximately 30% of participants expressed confidence in utilizing digital methods, a finding supported by literature emphasizing the clinical advantages of digital impressions in terms of accuracy and patient comfort [11, 12]. Nonetheless, this enthusiasm is tempered by practical constraints. Several studies have pointed out that the large tip size of scanners and the high associated costs limit their utility in neonatal CLP cases [13]. These limitations may partially explain the persistently low awareness and adoption rates identified in this study.

Barriers to adoption identified in this study further echo existing literature. A significant proportion (70%) cited a lack of training as a primary barrier, which is consistent with global trends highlighting deficits in digital dentistry education and exposure [14]. Additional barriers such as limited equipment access (58%) and high costs (42%) are reflective of persistent infrastructural challenges, particularly in resource-constrained settings [15, 16]. These findings reinforce the need for institutional and policy-level interventions to enhance infrastructure and access to technology. Furthermore, dentists with over 10 years of clinical experience and those with prior CLP-specific training demonstrated significantly higher knowledge and more favorable attitudes toward CLP impression techniques. These observations align with previous studies suggesting that professional experience and targeted education positively influence technology adoption [17, 18]. However, it is worth noting that some research contradicts this, proposing that years of experience alone may not guarantee openness to digital innovations, as younger dentists are often more adaptable to technological advances [16]. This study's observation of a significant urban-rural gap in attitudes (73±8 vs. 62±10, p<0.001) aligns with findings attributing such differences to disparities in resources and opportunities [7]. Nonetheless, these contrasts with other reports suggesting rural dentists may compensate for technological gaps through enhanced practical skills and adaptive clinical approaches [19]. The prevalence of training-related barriers (70%) clearly signals the need for structured and accessible continuing education programs. Such interventions have been shown to enhance knowledge, confidence and ultimately, the uptake of new technologies in clinical practice [20]. Addressing these gaps is imperative not only to improve individual practitioner competence but also to ensure equitable access to quality care for CLP patients, regardless of geographic location. These findings suggest that targeted training initiatives, particularly for rural practitioners and those with less clinical experience, could serve to bridge existing knowledge and confidence gaps. Additionally, policy measures aimed at subsidizing digital equipment or providing institutional access to advanced technologies could help alleviate financial and infrastructural barriers, as has been recommended in comparable low-resource settings [15]. The study acknowledges certain limitations. The potential for response bias exists due to the voluntary nature of participation and the possibility of self-selection among more motivated or digitally literate practitioners. Moreover, while the sample is geographically diverse across India, the findings may not be fully generalizable to other regions or countries with different healthcare infrastructures. Nonetheless, the inclusion of both urban and rural practitioners enhances the internal validity and relevance of the findings. Future research could focus on longitudinal studies evaluating the impact of targeted training programs on the actual adoption of digital technologies in CLP care. Additionally, incorporating clinical outcome measures could help establish clearer links between improved knowledge and practical benefits for patient care. Such studies would provide more robust evidence to guide educational and policy decisions. The findings of this study have direct implications for improving the quality of CLP care within general dental practice. By equipping general dentists with the necessary knowledge and skills, particularly in underserved areas, the broader dental community can contribute more effectively to the early and accurate management of CLP cases, thereby enhancing patient outcomes.

## Conclusion:

This study highlights significant shortcomings in general dental practitioners' knowledge and awareness of impression techniques for cleft lip and palate, especially regarding the use of digital intraoral scanning. Limited training opportunities, inadequate access to equipment, and financial limitations were the main obstacles, whereas practitioners in urban areas and those with prior CLP-specific training demonstrated better competence. Strengthening educational initiatives and improving access to resources are crucial to promoting the adoption of digital methods and enhancing the overall standard of CLP care.
